# B Cell Regulation in Autoimmune Diseases

**Published:** 2018

**Authors:** A. V. Sokolov, A. A. Shmidt, Y. A. Lomakin

**Affiliations:** Shemyakin-Ovchinnikov Institute of Bioorganic Chemistry RAS, Miklukho-Maklaya Str., 16/10, Moscow, 117997, Russia; Institute of Fundamental Medicine and Biology, Kazan Federal University, Kremlevskay Str., 18, Kazan, 420008, Russia

**Keywords:** Multiple sclerosis, systemic lupus erythematosus, rheumatoid arthritis, experimental autoimmune encephalomyelitis, Breg, regulatory B cells, IL-10, IL-35, CD19+CD24(hi)CD38(hi)

## Abstract

Antibody-independent B cell effector functions play an important role in the development and suppression of the immune response. An extensive body of data on cytokine regulation of the immune response by B lymphocytes has been accumulated over the past fifteen years. In this review, we focused on the mechanisms of inflammatory response suppression by subpopulations of regulatory B cells in health and autoimmune pathologies.

## INTRODUCTION

B cells are one of the central elements of humoral immunity. Traditionally, it had been believed that the main role of B cells lay in the production of antibodies, until their direct participation in cellular immunity was discovered later. B-lymphocytes are involved in T cell activation by antigen presentation, co-stimulation, and cytokine production; they affect antimicrobial protective mechanisms and inflammatory processes in the tissues of the body; they also act as regulatory cells that control both the cellular and humoral immune responses.

The existence of B cells capable of suppressing the immune response was first suggested as early as in the 1970s. Professor James Turk’s team found that removal of B cells from a pool of guinea pig splenocytes disabled the inhibition of delayed-type hypersensitivity (DTH) [1]. However, as it was not possible to characterize this observation from the molecular or biochemical point of view at that time, the studies were suspended. The regulatory properties of B cells were for the first time reliably described for experimental autoimmune encephalomyelitis (EAE), the animal model of multiple sclerosis, only 20 years later. Immunization of genetically modified mice with deletion of B lymphocytes (B10.PLµMT line) with a myelin basic protein (MBP) peptide led to the development of an acute and more severe form of EAE. The pathological process was uncontrollable, and there was no spontaneous remission characteristic of B10.PL mice producing mature B cells [2]. Over the past 10 years, much progress has been made in the study of immunosuppressive B cells. It has been found that regulatory B cells (Breg) can influence T cell differentiation, shifting it towards the regulatory phenotype [3]. Since then, the regulatory function of B-lymphocytes has been demonstrated in animal models of autoimmune colitis, rheumatoid arthritis, autoimmune diabetes, and systemic lupus erythematosus (SLE) [4-6].

## MECHANISMS OF REGULATORY B CELL FUNCTIONING

The very concept of regulatory B cells was first formulated by S. Fillatreau quite recently [4], when he described B cells (B10 cells) that produce interleukin-10 (IL-10), which can reduce clinical manifestations of EAE. IL-10 is one of the anti-inflammatory cytokines which regulate immune response and affect mainly antigen-presenting cells, reducing the expression of pro-inflammatory cytokines and the molecules involved in antigen presentation (MHC I, MHC II, adhesion molecules, etc.), and also inhibit the proliferation of CD4^+^ T lymphocytes [5]. Subsequent experimental removal of the population of B10 lymphocytes in mice also revealed a correlation with a decrease in the amount of Tregs, which was also associated with excessive proliferation of pro-inflammatory T cells after induction of the autoimmune response [6]. Bregs produce IL-10, and therethrough inhibit the differentiation of T helper type 1 (Th1) and T helper type 17 cells (Th17), decreasing the production of inflammatory cytokines by dendritic cells [7]. For this reason, production of IL-10 is the most extensively studied B cell regulatory mechanism and it is often applied to identify new Breg subpopulations. Nevertheless, other mechanisms could be used by Breg to control the development of an immune response, such as production of TGF-β (transforming growth factor-β), IL-35, IgM, IgG4, action on T lymphocytes through direct cell-to-cell contact, etc. (*Table*). At the same time, the regulation of immune processes using several simultaneous mechanisms is often observed, for example, by the production of IL-10 and TGF-β, both of which essentially inhibit the T cell response [8]. It was shown that lipopolysaccharide-activated B cells facilitate the apoptosis of CD4^+^ and inactivation of CD8^+^ effector T cells through the production of TGF-β despite an increased level of IL-10 expression [9, 10]. Particular attention should be paid to IL-35, another recently described key immunoregulatory cytokine produced by Bregs. Genetically modified mice, whose B cells do not express IL-35 subunits, developed acute EAE. In the case of inflammation caused by *Salmonella typhimurium*, the lack of IL-35 expression by B cells led to an increase in Th1 proliferation and increase in the amount of macrophages in the spleen [11]. Another independent study showed that IL-35-stimulated B cells-produced IL-35 and inhibited experimental uveitis under conditions of adoptive transfer [12]. An important role of Bregs in maintaining the equilibrium and functions of the type 1 natural killer cells (invariant natural killers, iNKT) required to maintain tolerance to autoantigens in autoimmune diseases has been proven [13].

**Table:** The functioning mechanisms of B regulatory cells

Regulatory mechanism	Effect	Experimentally validated in B cells of	
		Mouse	Human
IL-10 production	Inhibition of CD4^+^ T cell proliferation	✔[15]	✔[3]
	Inhibition of Th1 and Th17 differentiation	✔[4, 16]	✔[3, 17]
	Induction of regulatory T cell proliferation	✔[18–21]	✔
	Inhibition of TNF-α^1^ production by monocytes		✔[22]
	Inhibition of cytotoxic activity of T lymphocytes		✔[23]
	Inhibition of T follicular helper (T_FH_) and B cell differentiation		✔[24]
TGF-β production	Inhibition of Th1 and APC differentiation	✔[9, 11]	
	Induction of regulatory T cell proliferation	✔[24, 25]	✔[26]
	Regulation of macrophage activity	✔[27]	
	Inhibition of T follicular helper (T_FH_) and B cell differentiation		✔[24]
IL-35 production	Inhibition of activation of macrophages and pro-inflammatory T-lymphocytes	✔[11]	
IgM production	Induction of apoptotic bodies elimination	✔[28]	
	Suppression of allergic response of Th2	✔[29]	
Cell-to-cell contact	Inhibition of CD4^+^ T cell proliferation	✔[30,31]	✔[32]
GITRL^2^	Induction of regulatory T cell proliferation	✔[33]	
IgG4 production	Attenuation of complement system activation		✔[34]
BTLA expression^3^	Induction of regulatory T cell proliferation and activation	✔[35]	
	BTLA/HVEM^4^ interaction? Inhibition of T cell activation? Inhibition of B cell proliferation?		✔[36]
PD-L1 expression^5^	Suppression of inflammatory response by inhibiting T follicular helpers (T_FH_) and reducing antibody production	✔[37]	
	Induction of regulatory T cell proliferation?		✔[38]
	Inhibition of CD8^+^? Inhibition of CD4^+^? Inhibition of APC?		✔[23,39]

^1^ – TNF-α, tumor necrosis factor α;

^2^ – GITRL, glucocorticoid-induced tumor necrosis factor receptor-related ligand;

^3^ – BTLA, B and T lymphocyte attenuator;

^4^ – HVEM – herpes virus entry mediator;

^5^ – PD-L1 – programmed death-1-ligand.

As shown in *Table*, the aforementioned mechanisms primarily act on T lymphocyte subpopulations with proinflammatory properties by inhibiting their differentiation and development. However, other effects of Breg are also observed (e.g., attenuation of complementary system activation and elimination of apoptotic cells) that eventually also lead to a decrease in the intensity of the immune response [14].

Breg functioning involves CD40, TLR, B cell receptor, CD19, CD1d, etc. [14]. The membrane receptor CD40 activated by the corresponding ligand (CD40L present on the effector T cell membrane) can stimulate cascade reactions. Therefore, CD40 is involved in the development of memory B cells, the switching of immunoglobulin classes, and formation of germinal centers. Its participation in the functioning of regulatory B cells was shown in murine and human B lymphocytes. Activation of B cells in the presence of the ligand or activated T cells initiated the production of IL-10 and triggered a regeneration process in the case of EAE and, vice versa, suppression or elimination of the receptor (CD40^-/-^) disabled IL-10 synthesis.

**Fig. 1:**
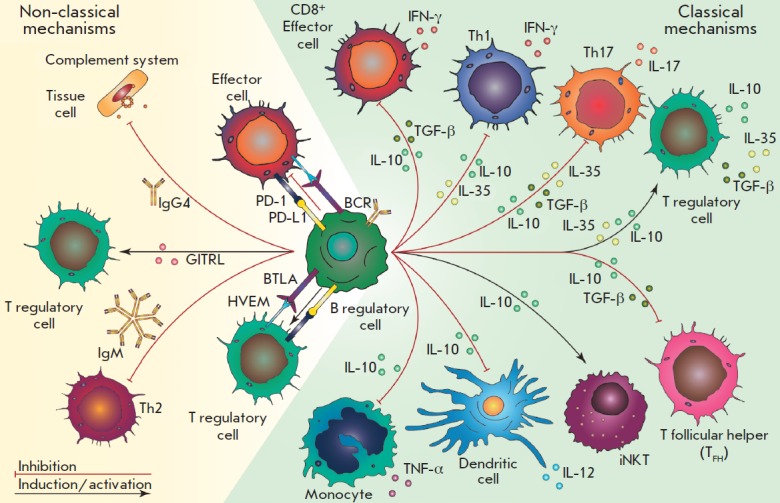
Mechanisms of regulatory B cell functioning and their impact on immune cells. Regulatory B cells produce anti-inflammatory cytokines that induce the formation of regulatory T cells and support invariant natural killers (iNKT), shown by black arrows. Breg-produced interleukins inhibit the differentiation of T follicular helpers, T helpers 1 and 17, inhibit the cytotoxic activity of T-lymphocytes (CD8^+^), and inhibit the production of pro-inflammatory cytokines by monocytes and dendritic cells (red arrows). Additionally, regulatory B cells reduce inflammation through direct cell contact, expression of B and T lymphocyte attenuators (BTLA), programmable death receptor ligands (PD-L1), production of IgM, IgG4, etc.

It is known that Toll-like receptors (TLRs) recognize a wide variety of molecular epitopes and play an important role in the signal transfer in innate and adoptive immunity. Stimulation of TLR with appropriate antigens increases the survival rate of mice in SLE and EAE models, as compared to a control group that did not receive the stimulating agent; this also results in a decrease in T cell proliferation and secretion of proinflammatory cytokines by these cells [40]. In *in vitro *studies on human splenic B cells and peripheral blood cells, stimulation with TLR antigens induced IL- 10 production and the highest impact involved stimulation with lipopolysaccharide and CpG (ligands TLR4 and TLR9, respectively) [22]. The role of BCR, CD19, and other surface B cell markers in the induction of a regulatory phenotype was also studied. It was shown that activation of receptors leads to IL-10 production, and to a decrease in the intensity of clinical manifestations of the investigated diseases in animal models. The absence of these molecules significantly reduces the ability of B cells to regulate immune responses [14]. Elevated levels of expression of B and T lymphocyte attenuators (BTLA) or the ligand of the programmable death receptor (PD-L1) in certain populations of regulatory B cells can lead to a decrease in the inflammatory response, due to the inhibition of effector T and B cells through an interaction with the HVEM or PD receptor, respectively [23, 35, 41]. The examples above demonstrate our improved understanding of the multiple roles of B regulatory cells, provided that Bregs can interact with many immune cells to suppress the immune response (*Fig. 1*). Abnormal functions and the amount of regulatory B cells are most often associated with autoimmune diseases. It is clear that how this subpopulation of lymphocytes functions must be strictly controlled by the body, starting from the recognition of proinflammatory signals by these cells in their microenvironment and ending with a strict control of their differentiation and development. Nevertheless, it remains unclear whether a Breg subpopulation is always present in the body or whether its development is induced by external signals. Although it is obvious that B lymphocytes perform many functions in both healthy and impaired immune systems, they play both pathological and protective roles in autoimmune processes, infections, and allergies [42]*. *

## PHENOTYPE AND ORIGIN OF REGULATORY B CELLS

When investigating B regulatory cells, it is also important to determine their phenotype. To date, many different subpopulations of Breg have been described, most of which are similar in both phenotype and functions. It is still unclear whether the differences observed between these subpopulations are due to the influence of the immunological environment or whether there are lines of B regulatory cells of different origins. In mice, the populations of regulatory B cells account for up to 5% of the total pool of B cells in the spleen and lymph nodes and their amount significantly increases with the development of inflammatory responses (e.g., EAE [43], collagen-induced arthritis [21], or helminthiasis [44]). There are three main subpopulations of regulatory B cells in mice: T2-MZP (transitional 2 marginal-zone precursor) CD19^+^CD21^high^CD23^highIg^M^high^ [31], CD19^+^CD5^+^CD1d^high^ [45], and Tim-1^+^ B cells [46]. In humans, B10 cells account for less than 1—2% of the total amount of B cells in the blood. Human Bregs include CD19^+^CD24^hi^CD38^hi^CD1d^hi^ and CD19^+^CD24^hi^CD27^+^ [22]. The relationship between the development and differentiation of these subpopulations is unknown. Although identification of IL-10 production was a good approach toward determining suppressor B cells, many of the surface marker molecules required for a more accurate characterization of the subpopulation can be differently expressed under conditions of immune response activation, making it difficult to study Bregs under various experimental conditions, which often alter the phenotype of Breg subtypes. This problem can be solved by means of identification of a Breg-specific transcription factor, which can be used to answer the question of whether these cells belong to the same developmental line. Currently, two models of Breg development can be suggested. According to the first one, regulatory B cells, like Treg, represent a separate B cell line with a specific set of factors of gene expression control responsible for their capability to suppress the immune response. The second theory is that B lymphocytes undergo phenotypic reconstructions in response to certain stimuli to suppress a local inflammation. Despite the studies in mice and humans, it has not yet been possible to identify a specific transcription factor. The inability to identify these markers, as well as the heterogeneity of Breg phenotypes, indicates that suppressor B cells are not a distinct developmental line: i.e., any B cell can be potentially differentiated into a regulatory one under the influence of external factors [8]. It was also shown that, along with previously described Breg subpopulations, plasmablasts can also suppress inflammatory responses. Mice lacking plasmablasts due to a genetic removal of the Irf4 and Prdm1 (Blimp1) transcription factors required for plasma cell differentiation developed acute EAE [7]. This is not the first case when B cells-producing antibodies also perform a regulatory function: CD138^+^ plasma cells, producing IL-10 and IL-35, suppressed pro-inflammatory responses in the case of EAE and a *Salmonella enterica *infection [11]. Moreover, splenic B10 cells that were differentiated into antibody-producing plasmablasts after stimulation both *in vivo *and *in vitro *have been described [47]. A relationship between CD19^+^CD24^hi^CD38^hi^ B cells performing regulatory functions and IL-10-secreting plasmablasts in humans has been suggested. This assumption suggests a similar differentiation vector, i.e. development into plasma cells, of Bregs in mice and humans. The idea that antibody-producing cells also regulate immune responses conflicts with the modern concept that plasma cells cause an inflammatory response, producing antibodies that are often pathogenic in the context of autoimmune diseases or allergies. Therefore, it is possible that a certain subpopulation of plasmablasts produces antibodies and, thus, supports the possibility of inflammatory response regulation. This assumption is supported by evidence that deficiency in Bcl6, the transcription factor required for B cell proliferation in germinal centers, does not affect the development of regulatory plasmablasts [7].

According to recent studies, immature B cells, mature B cells, and plasmablasts are able to differentiate into IL-10-producing Bregs in the body of mice and humans. This confirms the assumption that the B lymphocyte environment, rather than a specific transcription factor, is required for the differentiation of regulatory B cells. Thus, the search for the stimuli required for B cells to acquire regulatory functions becomes important in order to assess the origin of Bregs. However, it has recently been shown that differentiation of IL-10-producing regulatory B cells can be also induced by pro-inflammatory cytokines [8].

## THE ROLE OF REGULATORY B CELLS IN THE DEVELOPMENT OF AN INFLAMMATORY RESPONSE

There is strong evidence that inflammation leads to an increase in the amount of Bregs and their ability to suppress the immune response. It is known that they are present in naïve mice, but their amount increases with the development of some autoimmune diseases [31, 48]. Moreover, it was found that Bregs are involved in the suppression of inflammation in autoimmune pathologies. For example, the absence of Bregs in an animal model of MS results in the development of more severe and acute forms of EAE [4, 6]. Recently, it has been shown that the amount of regulatory B cells increases in response to the secretion of the proinflammatory cytokines IL-1β and IL-6 after induction of arthritis [49]. Secretion of these cytokines in mice with arthritis is controlled by bacteria in the intestine. Previously, the role of the microbiota had already been shown in the differentiation of pro-arthritogenic Th17 [50]. Mice grown in nonsterile conditions, whose B cells do not express IL-1R1 or IL-6R, develop acute arthritis [49]. Therefore, it can be assumed that Breg proliferation increases in response to IL-1β and IL-6 in order to prevent uncontrolled amplification of pro-inflammatory lymphocytes, such as Th17. Other inflammatory cytokines required for the differentiation of a Th17 phenotype, the IL-21 and granulocyte macrophage colony-stimulating factor (GM-CSF), together with IL-15, also play an important role in the development of Bregs [51, 52]. Various sources of cytokines that can enhance the production of IL-10 B cells have been identified. Myeloid cells of lymphatic vessels and spleen producing IL-6 and IL-1β are responsible for an increase in the amount of Bregs associated with arthritis, while CD4^+^ splenic T cells producing IL-21 activate Bregs in experimental arthritis models [49, 52]. On the other hand, administration of the anti-inflammatory cytokine IL-35 to mice increased the population of B cells expressing IL-10 and IL-35 and thereby suppressed the development of uveitis [53]. However, it should be taken into account that IL-35 is not expressed permanently, but is rather induced in response to inflammation [54].

Although these cytokines evidently play an important role in the proliferation of Bregs, it should be kept in mind that, during immune response development, B cell receptors (BCR) are also required for Breg induction. MD4 mice, whose BCR is specific to hen egg lysozyme (HEL), demonstrate impaired activation of Bregs during the development of EAE. It has been shown that chimeric animals with MD4 B cells incapable of IL-10 production develop a more severe form of EAE and are not capable of recovery [4]. Furthermore, MD4 B cells secrete less IL-10 and the amount of B10 cells themselves is lower than that in wild-type mice [45, 55]. The importance of correct recognition of BCR in Bregs is evidenced by the results obtained using mice with a specific deletion of the stromal interaction molecule 1 (STIM-1) and STIM-2 in B cells. These molecules are required for the regulation of the calcium inflow into the cytosol of B cells after BCR interaction with an antigen. Mice whose B lymphocytes lack STIM-1 and STIM-2 demonstrate a decrease in IL-10 production after stimulation with MOG (myelin oligodendrocyte glycoprotein) autoantigen [56]. These data show that antigen-specific recognition of the B cell receptor is important for the functioning and proliferation of Bregs. B cells can differentiate into regulatory or antibody-producing cells in response to B cell receptor recognition during the development of the immune response.

The significance of the inflammatory response in Breg differentiation raises the question of the place of their maturation. To date, most studies have investigated B cell populations in the spleen. However, in the case of colitis and EAE, Breg cells were also found in lymphatic vessels close to the inflammation site [7, 48]. Moreover, regulatory B cells can develop and gain the ability to suppress the immune response outside the spleen; namely, in the lymphatic vessels (in this instance, spleen removal does not affect their production) [7]. All these data support the theory that Breg induction is influenced by the inflammatory environment, which contradicts previously published results characterizing the spleen as the major regulatory B cell development site.

## B CELL REGULATION IN THE DEVELOPMENT OF AUTOIMMUNE PATHOLOGIES

**Multiple sclerosis (MS)**

The population of regulatory B cells also participates in the pathogenesis of MS, which holds a special place in the list of autoimmune pathologies and is one of the most socially and economically significant neurological diseases of our time. MS occurs mainly in middle-aged people and leads to an almost complete loss of working ability or, in the case of insufficiently effective and timely treatment, even death within 10–15 years. For a long time, the leading role in MS development was attributed to Tcell-mediated immunity. However, there is now extensive evidence of the important role of B cells in the pathogenesis of MS [57, 58]. Catalytic antibodies, hydrolyzing the myelin basic protein, one of the characteristic autoantigens in MS, were found in these patients [59, 60]. Although the etiology of MS is still not fully understood, special attention is paid to bacterial and viral infections, along with genetic predisposition, hormonal status, and climatic conditions as the factors associated with its development. It is believed that molecular mimicry and cross-reactivity can underlie the mechanisms of viral induction of the disease. In 2003, cross-reactive recognition of the nuclear antigen of the Epstein-Barr virus (EBNA) and the autoantigen peptide of the myelin basic protein (MBP) by the monoclonal T cell receptor was demonstrated [61]. Later on, cross-reactivity was also detected and validated in autoantibodies to the LMP1 protein of the Epstein-Barr virus and MBP [62, 63]. In the case of EAE, Bregs can inhibit autoimmune T cell responses by slowing the differentiation of the pro-inflammatory T helpers 1 specific to CNS autoantigens [57]. The absence of Bregs leads to an exacerbation of immune responses. As mentioned earlier, mice with EAE devoid of B10 cells develop an acute form of the disease without remission [4]. The regulatory functions of IL-10-producing B cells were confirmed by the results of the study, where adaptive transfer of wild-type B cells reduced the severity of EAE manifestations in contrast to a transfer of IL-10-/- B lymphocytes from μMT mice. In that experiment, B cells from the first group of mice produced IL-10. Recently, the relationship between B and T regulatory cells in the development of EAE pathology has been characterized [43]. Indeed, adoptively transferred B10-cells directly affected the pathogenesis of EAE, as in the study by M. Yang [64], and their amount increased in the spleen, but not in the central nervous system, which is in agreement with the idea that they possess regulatory functions. Moreover, the transfer of antigen-activated B10 cells into wild-type mice strongly inhibited EAE induction, but B10 lymphocytes could not inhibit further EAE progression. At the same time, the amount of regulatory T cells in the central nervous system significantly increased with the development of the disease and this process influenced the course of EAE at the late stages. These data suggest that Bregs play a key role at the early stages of the disease, while Tregs perform regulatory functions in further development of the disease.

The EAE model showed that regulatory B cells are involved in the development of the pathological process. The levels of IL-10 production by peripheral blood B lymphocytes in MS patients were first determined in 2007 [65]. A significantly lower level of IL-10 production by B cells stimulated in the presence of the CD40 ligand was found in groups with relapsing-remitting and secondary-progressive MS compared to healthy donors. A similar effect was observed in the case of B cell stimulation with CpG [66]. Therefore, impaired IL- 10 production and the functions of regulatory B cells from the peripheral blood of MS patients have been established. Apart from IL-10 production, regulatory B cells are involved in the development of MS by the production of IL-35 and TGF-β, and they can also enhance Foxp3 and CTLA-4 expression in regulatory T cells, as a result of direct cell contact [11, 32].

**Fig. 2:**
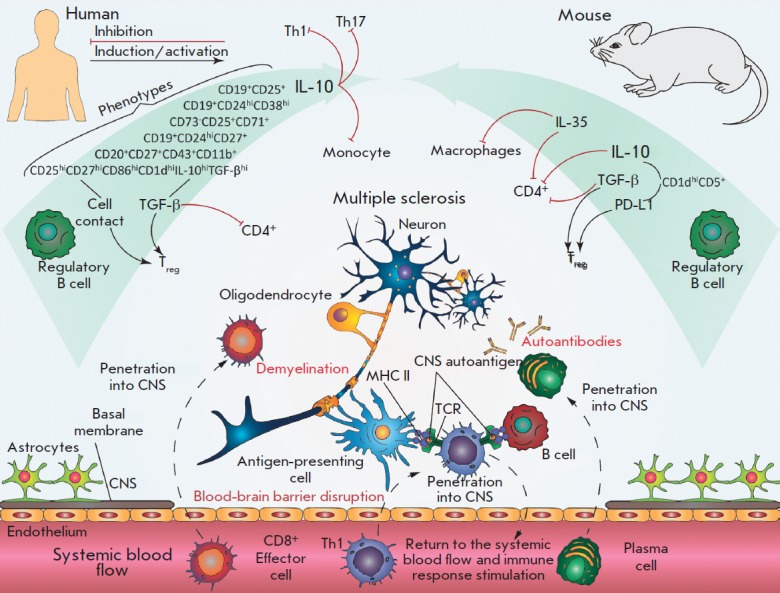
Participation of regulatory B cells in the pathogenesis of multiple sclerosis. During the disease, Bregs can suppress the development of the autoimmune reaction, along with production of autoantibodies, autoantigen presentation, and activation of the T cell response. Various subpopulations of regulatory B cells with corresponding surface markers were identified in murine models and MS patients. In most cases, the immunosuppressive function of Breg is performed by the production of IL-10, IL-35, TGF-β, and direct cell-cell interactions

Thus, B cells can perform dual functions in the development of the demyelination process (possibly both positive and negative effects on immune responses), but their role in the pathogenesis of MS is well-traceable (*Fig. 2*).

**Systemic lupus erythematosus (SLE)**

Systemic lupus erythematosus is a chronic autoimmune disease of connective tissue characterized by a wide range of clinical manifestations. The danger of SLE is associated with the possibility of simultaneous involvement of many vital organs, which leads either to death or chronic health deterioration [67]. Increase in the titer of autoreactive antibodies, such as anti-DNA, anti-nuclear, anti-Ro, anti-La, anti-Sm, anti-RNP, and anti-phospholipid antibodies, is observed at different stages of the disease, often before the onset of clinical symptoms [68, 69]. In this case, detection of autoreactive antibodies is not considered as a sufficient criterion of disease onset, and, therefore, other factors, genetic and exogenous ones, may play an important role [67]. The causes of SLE are still unknown, although the current view that apoptosis largely contributes to the pathogenesis explains why the immune system reacts primarily to internal antigens. Autoantigens are released by cells that have undergone apoptosis and necrosis. The disorders in the elimination of apoptotic cells described in patients with this disease can lead to their abnormal ingestion by macrophages, which, in turn, provide intracellular antigens to T and B cells, thereby triggering an autoimmune process [70]. The cytokine status of the organism also affects the development of the disease. Most patients with an active form of SLE demonstrate increased expression of interferon-alpha (IFN-α), which can enhance the function of antigen-presenting cells and activation of T cells [71].

**Fig. 3:**
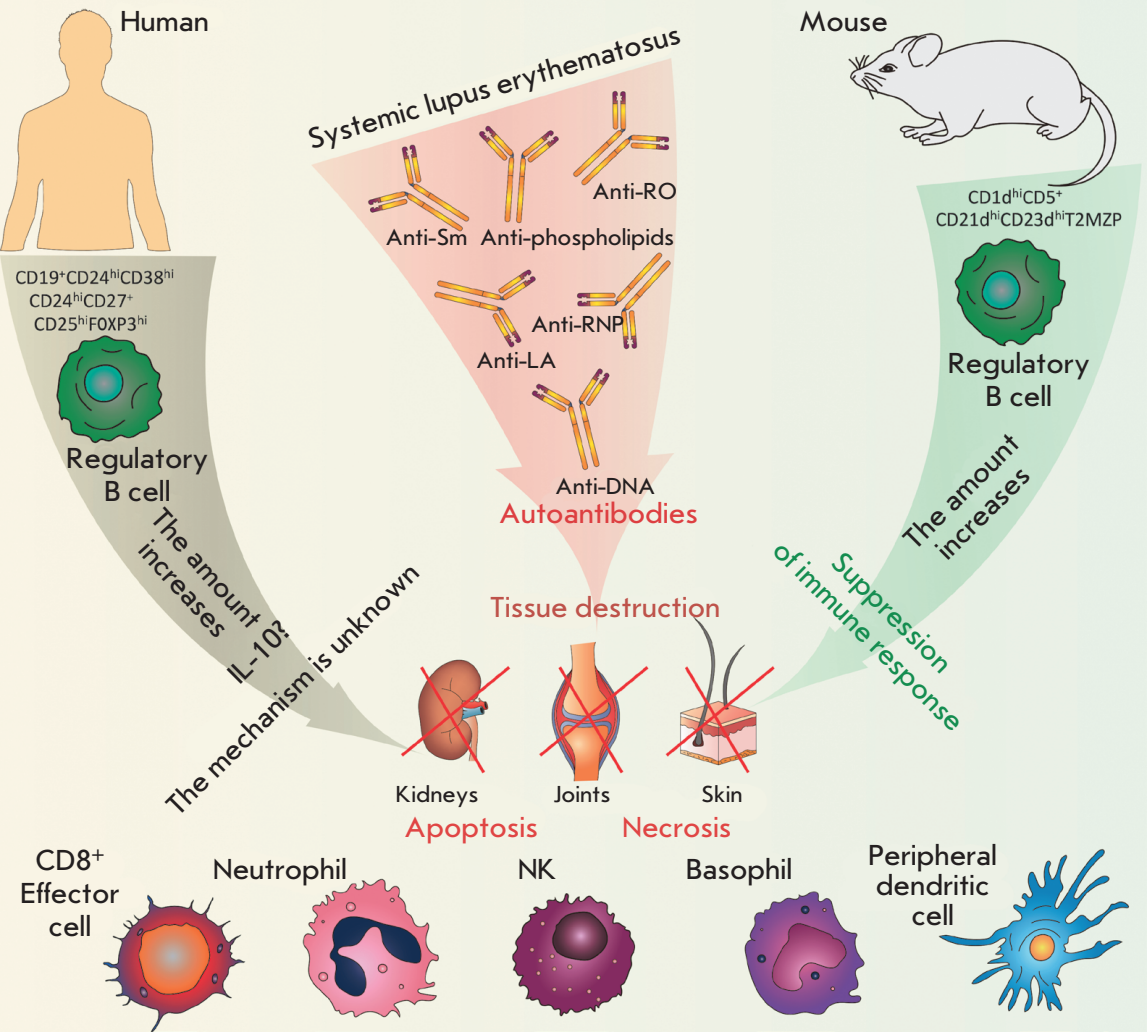
Participation of regulatory B cells in the development of systemic lupus erythematosus. During the disease, B cells participate in the regulation of the autoimmune inflammation, along with the production of autoantibodies to nuclear autoantigens. Various subpopulations of regulatory B cells with corresponding surface markers were identified in murine models and patients with SLE, whose number increases with the course of the disease. An apparent protective role of Bregs was shown in animal models. In patients with SLE, the mechanism is not fully understood at the moment

It is known that regulatory B cells are important for SLE suppression (*Fig. 3*). It was shown in murine models that two independent populations of regulatory B cells, CD1d^hi^CD5^+^ and CD21^hi^CD23^hi^ T2 MZP, play a protective role in the development of the disease, and that their activation contributes to the survival of animals [20, 72]. At the same time, the question of the participation of regulatory B cells in the pathogenesis of SLE in humans remains open. It was shown that the amount of regulatory B cells increases with the development of the pathology [22] and even correlates with the severity of the disease [73]. However, the anti-inflammatory function of the CD19^+^CD24^hi^CD38^hi^ population worsens as the disease progresses [17].

**Rheumatoid arthritis (RA)**

Rheumatoid arthritis is a disease with unknown etiology that manifests itself in connective tissue and joint impairment resulting from an autoimmune inflammatory response. The pathogenesis of rheumatoid arthritis involves a lot of immune cells, as well as various cytokines and arachidonic acid metabolites. The role of B cells in this disease is associated primarily with the production of autoantibodies to the Fc-domain of IgG (rheumatoid factors), as well as autoantibodies to the cyclic citrulline peptide, carbamylated proteins, etc. [74, 75]. For a long time, the role of regulatory B cells remained insufficiently studied.

IL-10, IL-35, and TGF-β are the main effector molecules of regulatory B cells in the development of RA. IL-10 is a typical anti-inflammatory cytokine: its influence on the course of rheumatoid arthritis is considered as favorable, since it inhibits the action of autoimmune Th17 and reduces IL-17 production by immune cells, preventing joint destruction [76-79]. IL-35 is another immunosuppressive cytokine. However, there are controversial data on its impact on the course of rheumatoid arthritis. Some studies have demonstrated a protective effect of IL-35 on the development of RA due to a decrease in IL-17 and IFN-γ production, as well as inhibition of VEGF [80, 81]. Other studies suggest that IL-35 has a pro-inflammatory effect and is directly involved in the pathogenesis of this disease. Furthermore, its plasma level decreases during treatment [82, 83]. The effect of TGF-β cannot be referred to as totally immunosuppressive and favorable to RA, although this cytokine is characteristic, for example, of regulatory T cells and enhances the expression of their main regulator, the FOXP3 transcription factor [84]. A significant increase in the level of TGF-β was found in animal models of RA (collagen-induced arthritis in mice and rats immunized with type 2 collagen, as well as TNF-α transgenic mice) compared to non-immunized control animals. Moreover, the increase in the level of this cytokine was accompanied by the involvement and incorrect differentiation of mesenchymal stem cells and pre-osteoblasts in the subchondral area of the bone marrow, which contributed to joint degeneration. At the same time, inhibition of TGF-β reduced the amount of these cells in this area, reduced chondrocyte hypertrophy, and slowed down joint degeneration [85]. However, in a similar study, inhibition of TGF-β in a mouse model of RA (collagen-induced arthritis) had virtually no effect. In this case, increased activity of this cytokine was observed in the lymphoid cells of tissue samples from RA patients [86]. Parallel studies showed that RA patients have a lower level of CD19(+)TGFβ(+) Bregs than healthy donors [87].

**Fig. 4:**
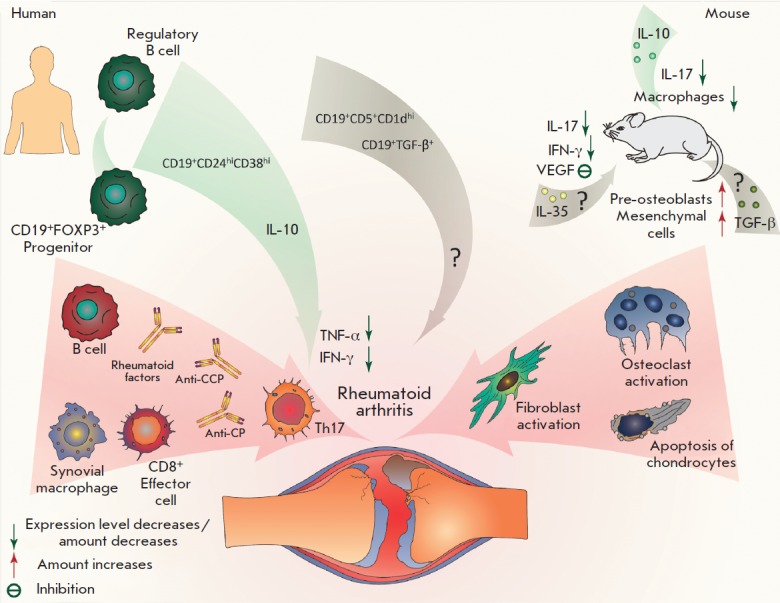
Participation of regulatory B cells in the development of rheumatoid arthritis. During the disease, B cells participate in the regulation of the autoimmune inflammation, along with the production of autoantibodies. Three main subpopulations of regulatory B cells were discovered in RA patients. CD19^+^CD24^hi^CD38^hi^ demonstrated a suppression of the inflammatory response by inhibiting Th17 activity and reducing the level of IFN-γ and TNF-α in a IL-10-dependent manner. The mechanism and role of the CD19^+^CD5^+^CD1dhi and CD19^+^TGF-β^+^ subpopulations in the development of RA has not yet been clearly identified. An apparent protective role of IL-10 was shown in animal models. Participation of IL-35 and TGF-β is in question

Evaluation of a direct impact of regulatory B cells on the course of rheumatoid arthritis development is challenging, since RA, like other autoimmune diseases, is characterized by the existence of Breg populations that differ in surface markers. In this case, it seems that phenotypically different Bregs can perform different functions in the pathogenesis of RA (*Fig. 4*). It was shown that the level of CD19^+^CD5^+^CD1d^hi^ decreases in RA patients. In this case, the granzyme-producing B cells CD19^+^CD5^+^GzmB^+^ may be involved in the pathogenesis of this disease [88]. It was found that the level of IL-10^+^ B cells in patients with rheumatoid arthritis remains the same as in healthy donors. However, induction of these cells from CD19^+^ B-lymphocytes sampled from patients using CpG deoxyoligonucleotide and CD40L was easier than in healthy donors. A negative correlation was found between the amount of induced IL-10^+^ B cells and the severity of the disease according to the DAS28 index (disease activity score in 28 joints) [89]. An analysis of the potential precursors of IL-10^+^ B cells (CD19^+^TGF-β^+^ and CD19^+^FOXP3^+^ populations) showed a decrease in the number of both populations in patients with rheumatoid arthritis. However, only the FOXP3^+^ population showed a negative correlation with the severity of the disease [87]. It was also shown that IL-10^+^ B cells cannot be considered as a separate population and that the number of these cells inversely correlates with the severity of the disease, especially during the first 5 years after diagnosis [90]. CD19^+^CD24^hi^CD38^hi^ B cells were found to inhibit the production of IFN-γ and TNF-α CD4^+^ T cells. Moreover, CD19^+^CD24^hi^CD38^hi^ hampers the differentiation of CD4^+^ T cells into the Th1 and Th17 associated with rheumatoid arthritis. The number of regulatory B cells of this phenotype is reduced in the active phase of the disease [3]. The study of CD19^+^CD24^hi^CD38^hi^ B cells provided conflicting results. The level of these cells is high in patients with rheumatoid arthritis, which, again, is indicative of a variety of regulatory B cells and their various functions [91]. Note that increasing concentration of cells cannot be unambiguously regarded as a signal that they contribute to the progression of the disease, since this can be interpreted as a compensatory reaction by the organism. It is assumed that IL-10^+^ B cells are part of the population of CD19^+^CD24^hi^CD38^hi^ B cells, and these data agree with earlier results [17, 91]. When comparing the population of CD19^+^CD24^hi^CD38^hi^ with all CD19^+^ B cells, the number of IL-10-producing cells is higher in this population [17, 91]. No correlation between the level of IL-10^+^ B cells and the concentration of proinflammatory cytokines in the serum of patients with rheumatoid arthritis was found, but the number of these cells is inversely proportional to the duration of the symptoms and the number of affected (swollen) joints. Note that heterogeneity of IL-10^+^ B cells was detected, some of which were characterized by a lower production of IL-10 and weaker inhibition of CD3^+^ lymphocyte proliferation [91].

The general picture that emerged during the studies of regulatory B cells in RA patients is rather indicative of their immunosuppressive role. However, taking into account the results of those aforementioned studies, it can be concluded that regulatory B cells are highly heterogeneous (even within the same population) and do not always identically influence the course of rheumatoid arthritis. Additional studies will provide accurate information about the functions of regulatory B cells in the pathogenesis of rheumatoid arthritis. Note that the evaluation of the impact of these cells is hampered not only by their heterogeneity, but also by their small amount and the complex action of their effector molecules.

## CONCLUSION

Over the past decade, the key role of B cell regulatory elements in maintaining immunotolerance, controlling, and suppressing the inflammatory response has been confirmed in numerous independent studies. Some disparity in the data and the absence of an unambiguous phenotypic portrait of these cells are largely due to the great heterogeneity of their subpopulations. Despite many questions about the exact regulation mechanism, it is obvious that abnormal amounts and functioning of Breg can lead to a number of immunological pathologies: in particular cancer, autoimmune, and chronic infectious diseases. Therefore, further investigation of the role of the B cell regulation of the inflammatory response will further not only our understanding of the etiology of autoimmune pathologies, but also the development of approaches to the therapeutic use of regulatory B cells.

## Abbreviations

EAE: experimental autoimmune encephalomyelitis
APC: antigen-presenting cells
MHC: major histocompatibility complex
IL: interleukin
MS: multiple sclerosis
RA: rheumatoid arthritis
Breg: regulatory B cell
Treg: regulatory T cell
SLE: systemic lupus erythematosus
CNS: central nervous system
